# Discovery of novel non-ATP competitive FGFR1 inhibitors and evaluation of their anti-tumor activity in non-small cell lung cancer *in vitro* and *in vivo*

**DOI:** 10.18632/oncotarget.2122

**Published:** 2014-06-20

**Authors:** Jianzhang Wu, Jiansong Ji, Bixia Weng, Peihong Qiu, Karvannan Kanchana, Tao Wei, Yi Wang, Yuepiao Cai, Xiaokun Li, Guang Liang

**Affiliations:** ^1^ Chemical Biology Research Center, School of Pharmaceutical Sciences, WenzhouMedical Universtiy, Wenzhou zhejiang China; ^2^ Department of Interventional Radiology, The Fifth Affiliated Hospital of Wenzhou Medical University, Lishui, Zhejiang, China

**Keywords:** Fibroblast growth factor receptor 1, non-small-cell lung cancer, non-ATP competitive FGFR1 inhibitors, NDGA, Anti-cancer activity

## Abstract

Accumulating evidence suggests that high expression of FGFR1 is closely related to the development of lung cancer especially in non-small cell lung cancers (NSCLC), to which non-ATP competitive inhibitors represent an effective therapeutical approach due to their good specificity. Herein, a series of NDGA analogues with the framework of bisaryl-1,4-dien-3-one as novel FGFR1 inhibitors have been designed and screened. Among them Aea4 and Aea25 showed strong FGFR1 ‵inhibition and high selectivity over other receptor kinases. The kinase inhibitory assay in different ATP concentrations and computer-assistant molecular docking showed that the FGFR1 inhibition mode of both Aea4 and Aea25 was non-ATP-competitive. The in vitro and *in vivo* study on anticancer efficacy of Aea4 and Aea25 against non-small cell lung cancer involves inhibition of cell proliferation, apoptosis induction and cell cycle arrest with no toxicity. Thus, these two novel non-ATP competitive inhibitors derived from NDGA may have a great therapeutic potential in the treatment of NSCLC. This work also provides a structural lead for the design of new non-ATP-competitive FGFR1 inhibitors.

## INTRODUCTION

Non–small-cell lung cancer is the leading cause of death from cancer. Approximately 85-90% of newly diagnosed lung cancers are non-small cell lung cancers (NSCLCs). Despite recent advances in treatments for the disease, the currently available systemic therapies for NSCLC have limited efficacy, indicating the need for innovative treatment strategies [1]. At present the research on receptor tyrosine kinase (RTKs) inhibitors is a hotspot of NSCLC treatment. The abnormal activation of receptor tyrosine kinase (RTK) in the pathology of many cancers has called attention to this family of receptors, which include widely studied epidermal growth factor receptor (EGFR) [2], fibroblast epidermal growth factor (FGFR1) [3], and vascular endothelial growth factor receptor (VEGFR) [4], in addition to other members [5, 6].

Fibroblast growth factor receptor (FGFR) family is a key lineage of the receptor tyrosine kinase superfamily with four distinct isoforms (FGFR1–4) found across several tissue types and expressed to different extents under varying conditions [7]. The high expression of FGFR1 is closely related to the development of lung cancer especially in NSCLC, and it plays a crucial role in tumor cell proliferation, angiogenesis, migration and survival [8, 9]). FGFR inhibition can reduce proliferation and induce cell death in a variety of in vitro and in vivo tumor models harboring FGFR aberrations and a growing number of research groups have selected FGFRs as targets for anticancer drug development [10, 11]. Silencing FGFR1 by siRNA or FGFR kinase inhibitors can both inhibit the growth of lung cancer cells effectively and block lung cancers in mice [12].

Targeting and inhibiting the FGFR signaling pathway is one evolving anticancer therapy with potential efficacy because FGFRs have several pockets that allow binding of small molecules for the inactivation of the kinase activity [13, 14]. However, the outcomes of recent phase II clinical trials with two inhibitors AZD4547 and BGJ398 against FGFR1 have been unsatisfactory (www.clinicaltrial.gov), and many small molecular tyrosine kinase inhibitors (TKI) such as SU5402, PD173074, TKI-258, and SU6668 have been are failed to enter clinical research or clinical use [13, 15]. These inhibitors were rationally designed based on competitive and reversible inhibition of the ATP-binding domain of FGFR1. It acts on the ATP-binding site which is deeply conservative among all the tyrosine kinases. Therefore they exhibit poor selectivity profile, severe side effects, and the drug potency is easily affected by the high intracellular ATP concentration [15]. Hence, the focus has been turning to the new type of RTKs inhibitors with the better targeted selectivity with low toxic effect [15-17]. The non-ATP competitive inhibitors which bind to the new sites instead of the ATP binding site may possess the superior selectivity [16]. Till date, only five non-ATP-competetive FGFR inhibitors have been identified, which includes nordihydroguaiaretic acid (NDGA, for FGFR3) [18], NF449 (for FGFR3) [19], ARQ069 (for FGFR1&2) [14], and recently reported A114 and A117 (for FGFR1) [20].

**Figure 1:**
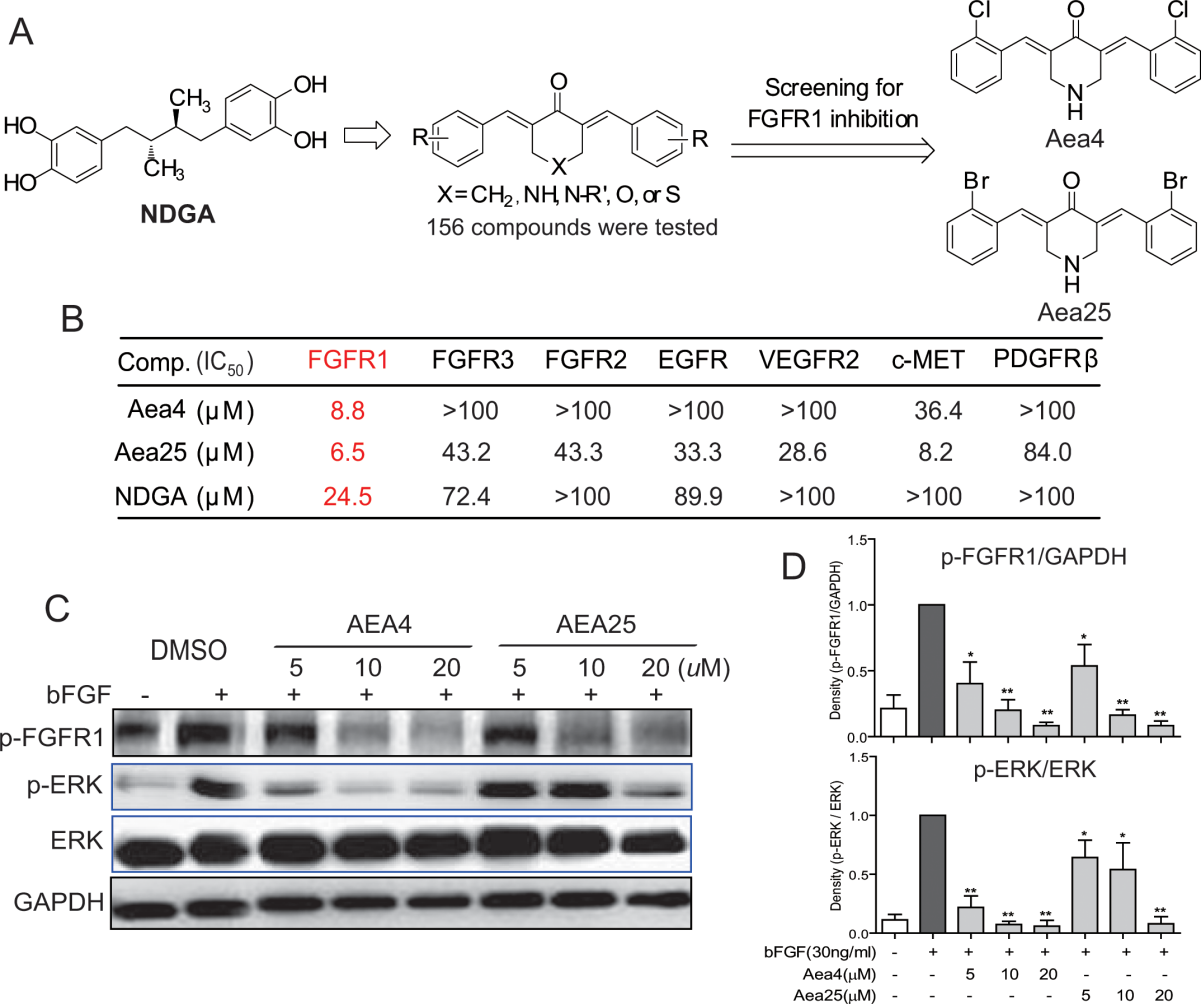
NDGA analogs Aea4 and Aea25 inhibited FGFR1 activities (A) The profile of design and FGFR1 kinase inhibition assay of NDGA analogs. (B) Aea4 and Aea25 selectively inhibit FGFR1. Compounds were performed with caliper mobility shift assay for RTK inhibition, and the IC_50_ values were calculated using conversion rates. The data were shown as a mean of 3-5 independent tests. (C and D) FGFR1 over-expressing 293 cells were pretreated with compounds at indicated concentrations or vehicle (0.1% DMSO), respectively. Then, cells were stimulated with bFGF (30 ng/mL) for 10 min, and the phosphorylation level of FGFR1 and ERK in cell lysates was measured by western blot analysis. The figures were representative of 3 separate experiments (C). The column figure shows the normalized optical density as a percentage of total protein control (D). Statistical significance relative to bFGF alone group was expressed, * *P* <0.05, ** *P* < 0.01.

Nordihydroguaiaretic acid (NDGA), the main metabolite of the creosote bush, has shown to have promising applications in the treatment of multiple diseases [21]. NDGA was recently shown to inhibit several receptor tyrosine kinases, including insulin-like growth factor (IGF-1) receptor [22, 23], c-erbB2/HER2/neu (HER2/neu) receptor [23, 24], and fibroblast growth factor receptor-3 [18]. NDGA likely exerts its anti-cancer activity in part through inhibiting these tumor-promoting receptor tyrosine kinases. In addition, as a natural compound, NDGA showed nontoxicity and could be used as a leading compound for further structural modification and medicinal chemistry research. In the present study, we identified two novel non-ATP-competitive FGFR1 inhibitors (Aea4 and Aea25, in Figure 1A) among 156 synthetic NDGA analogs and described their *in vitro* and *in vivo* anti-cancer activity.

## RESULTS

### Aea4 and Aea25 inhibit the activity of FGFR1 selectively

NDGA has been reported to inhibit an activated FGFR3 mutant and block downstream signaling in multiple myeloma cells [18]. In our previous work, we found that the IC_50_ values of NDGA against FGFR1 and FGFR3 were 24.5 and 72.4 μM, respectively, indicating that NDGA exhibits better inhibitory activity against FGFR1 and FGFR3 (Figure 1B). Therefore, using NDGA as a leading compound, we designed and synthesized 156 new NDGA analogues with the framework of bisaryl-1,4-dien-3-one(Figure 1A), and screened the FGFR1 kinase inhibitory activity of analogues by Caliper Mobility Shift Assay. Out of 156 compounds, the FGFR1 kinase inhibitory activities of Aea4 and Aea25 (IC_50_=6.5μM and 8.8μM, respectively) were better than NDGA (Figure 1B). In order to verify the specificity, we further determined the inhibitions of Aea4 and Aea25 against other RTKs including FGFR2, FGFR3, cMET, EGFR, KDR, and PDGFRb. Besides cMET, Aea25 displayed a much lower activity against other RTKs compared to that of FGFR1. Aea4 had a weaker inhibition against cMET, and had no obvious inhibitory activity against other RTKs. Therefore, Aea4 and Aea25, especially Aea4 exhibited high specificity to FGFR1.

### Aea4 and Aea25 restrain the FGFR1 effectively in HEK293 cells

The inhibitory activity of Aea4 and Aea25 on FGFR1 activation was tested on FGFR1-overexpressing HEK293 cells, using bFGF (30ng/ml) as an inducer. As shown in the Figure 1C&D, bFGF(30ng/mL)significantly induced the phosphorylation of FGFR1 and ERK in HEK293 cells treated with DMSO whereas pretreatment with Aea4 and Aea25 inhibited their phosphorylation of FGFR1 and ERK in a dose-dependent manner.

### Aea4 and Aea25 inhibit the FGFR1 kinase in an ATP independent manner

**Figure 2:**
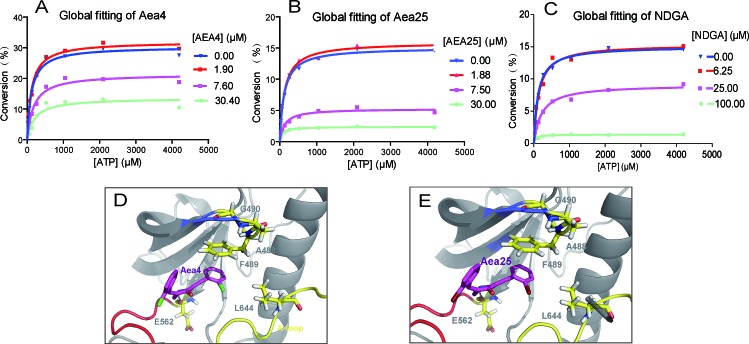
Aea4 and Aea25 inhibit FGFR1 in an ATP-noncompetitive manner ATP-competitive kinase assay of compounds Aea4 (A), Aea25 (B), and NDGA (C) with FGFR1 was carried out through caliper mobility shift assay. The conversion data were fitted with Graphpad for global fitting, using “mixed model inhibition.” (D-E) Molecular docking simulation of Aea4 (D) and Aea25 (E) with FGFR1 protein was conducted with Tripos' molecular modeling package, Sybyl-x.v1.1.083.

Due to their good specificity towards FGFR1, we speculate that Aea4 and Aea25 may be non-ATP-competitive inhibitors. To determine the mode of action of these two FGFR1 inhibitors, caliper mobility shift assay was used to determine the competitive relationship between ATP and compounds. The results were shown in the Figure 2A-C. The increased concentration of ATP did not affect the rate of FGFR1 substrate phosphorylation at the various concentrations of Aea4, Aea25, and NDGA (Figure 2A-C). In other words, the activity against FGFR1 kinase of compounds did not depend on the concentration of ATP. Thus, Aea4 and Aea25 suppressed FGFR1 in a mode of ATP independent manner consistent with the inhibition of the leading compound NDGA.

Next, we conduct the predictive investigation of their binding modes using a computer-assistant molecular docking. A previously-described ligand-receptor complex crystal structure (PDB Code: 3RHX) of FGFR1 with ARQ069, a non-ATP-competitive FGFR1 inhibitor, was used as a reference in the docking study [14]. We docked Aea4 and Aea25 with the inactive conformation of FGFR1 kinase. Figures 2D and 2E show that these two compounds are located on the same hydrophobic region of FGFR1 pocket and are encompassed by the residues E562, L644, and F489. The amino nitrogen in piperidone moiety of Aea4/Aea25 makes a hydrogen-bond interaction with E562. Importantly, the comparison of the modeling of FGFR1-Aea4/Aea25 with previously-solved FGFR1-ARQ069 showed a similar mode for Aea4/Aea25 and ARQ069 binding to the inactive and auto-inhibitory conformation of FGFR1 [14]. Figures 2D and 2E showed that the phenylalanine (F489) of the glycine-rich loop made a downward movement and established a hydrophobic interaction with the phenyl ring of Aea4/Aea25. The interaction between Aea4/Aea25 and the phenyl ring of F489 serves as an anchor to stabilize the conformation of the glycine-rich loop and contributes to the preference of Aea4/Aea25 for the inactive conformation of FGFR1.

### Aea4 and Aea25 inhibited proliferation of H460 cells and induced apoptosis

**Figure 3:**
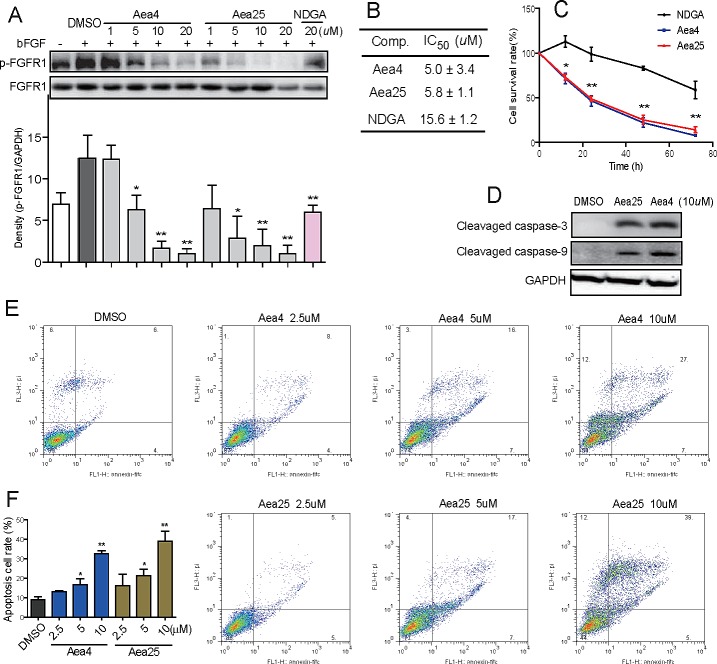
Aea4 and Aea25 inhibited proliferation and induced apoptosis in H460 cells (A) H460 cells were pretreated with compounds at indicated concentrations, followed by the treatment with bFGF (30 ng/mL) for 10 min. The phosphorylation level of FGFR1 in cell lysates was measured by western blot analysis. The column figures show the normalized optical density as a percentage of total FGFR1 control. (B) H460 cells were treated with compounds at concentrations (0.74, 2.22, 6.67, 20, and 60 μM) for 72 h. The MTT assay gives the respective IC_50_ values of compounds. (C) The time-course cell survival profile of H460 cells treated by compounds at 20 μM was detected through an MTT assay. (D) Effects of Aea4 and Aea25 on caspase activation in H460 cells. H460 cells were harvested and lysated after incubated with Aea4 (10 μM) and Aea25 (10 μM) for 12 h. The levels of cleaved caspase-3 and cleaved caspase-9 were determined by western blot analysis. (E) Aea4 and Aea25 induced cell apoptosis in H460 cells. H460 cells were treated with Aea4 and Aea25 at indicated concentrations for 24 h, and then stained with Annexin V and PI, followed by detection using flow cytometry. The figures were representative of three separate experiments. (F) The column figure shows the apoptotic cell rate detected by flow cytometer. (* *P* <0.05, ** *P* < 0.01).

In the development of cancer, FGFR1 is required for the proliferation of variety of cancer cell in which FGFR1 is amplified. Previously, we have found that the NSCLC H460 cell has the relative high expression of FGFR1 (Data not shown). Using NDGA as the positive control, the inhibitory activity of Aea4 and Aea25 against FGFR1 kinase in H460 cells were tested. Figure 3A showed that bFGF(30ng/ml)induced the phosphorylation of FGFR1 significantly, and Aea4 and Aea25 inhibited this effect in a dose-dependent fashion. They all exhibited preferable activity at the concentration of 5μM. The activity of Aea4 and Aea25 exhibited better inhibition than compared to the NDGA at the same concentration (20μM). Figure 3B shows that the IC_50_ values of Aea4 and Aea25 against H460 cells were 5.0μM and 5.8μΜ, respectively. Subsequently, time course experiment was performed using MTT assay. The H460 cells were incubated with Aea4, Aea25, or NDGA at a concentration of 20μM for 0, 12, 24, 48, and 72 hours, respectively. The result in Figure 3C suggested that Aea4 and Aea25 can inhibit cell proliferation and increase cell death of H460 time-dependently. The anti-proliferation activity of Aea4 and Aea25 was significantly higher than NDGA.

The activation of cleaved caspase-3 and cleaved caspase-9 were determined to understand whether compounds Aea4 and Aea25 can increase the apoptosis cascade via caspase activation by western blotting analysis. Figure 3D showed that the expression of cleaved caspase 3 and 9 was significantly increased in the H460 cells treated with Aea4 or Aea25 at 10μM for 12 hours. In addition, the flow cytometry apoptosis detection was performed to further confirm Aea4 and Aea25 -induced apoptosis in H460 cell line. As shown in Figures 3E & 3F, the rate of apoptotic cell rate in H460 cells was increased after the treatment with Aea4 or Aea25 at indicated concentrations(2.5, 5, 10μM)for 12 hours. These data suggested that Aea4 and Aea25 induced H460 cell apoptosis in a dose-dependent manner.

### Aea4 and Aea25 inhibit H460 tumor growth in xenograft models potently

**Figure 4:**
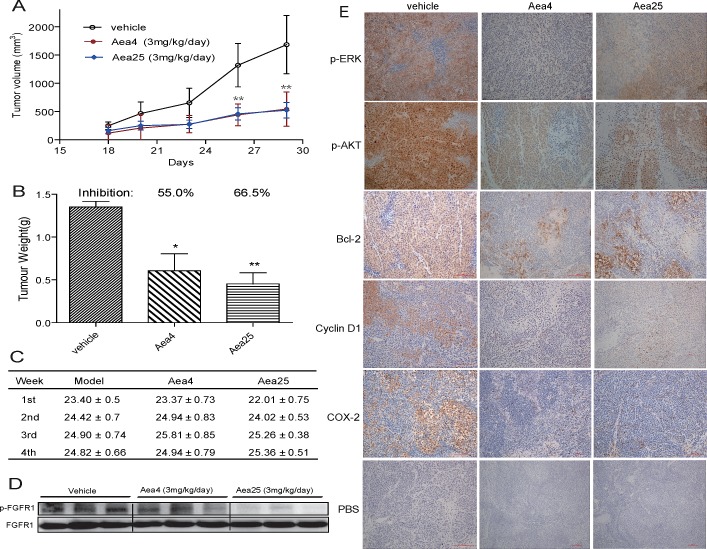
Anti-tumor effects of compounds Aea4 and Aea25 in H460 xenograft models Xenografts were established in nude mice. After two days, the mice were treated with liposome vehicle (once daily, i.p.), Aea4 (once daily, i.p., 3 mg/kg/d) or Aea25 (once daily, i.p., 3 mg/kg/d) for 28 days (n=10 in each group). (A) Tumor volume (mm^3^) and (B) Tumor weight (g) were recorded (* *P* <0.05, ** *P* < 0.01). (C) Body weight of each mice was recorded. (D) pFGFR1 expressions in tumor tissues were detected by Western Blot with FGFR1 as internal control. (E) The levels of p-ERK, p-AKT, Bcl-2, CyclinD1, and COX-2 in tumor tissues were detected by immunohistochemical staining. Representative pictures are shown.

Further, to assess the in vivo anti-tumor potential of Aea4 and Aea25, xenograft tumor models were established by inoculating H460 cells in BALB/cA nude mice. On the second day after inoculation, the mice were given intraperitoneal injection of Aea4 and Aea25 for 28 days (3 mg/kg/d). The tumor size was determined, and the tumor growth curve was drawn after 18th day. Figure 4A and 4B shows the tumor weight and tumor inhibition rate. Our data suggested that compared to the model group, administration of Aea4 or Aea25 resulted in reduction in tumor volume at the 20th day, and significant reduction in tumor volume at the 28th day (Figure 4A). The tumor weight was significantly decreased in Aea4- and Aea25-treated group with the inhibition rate of 55.0% and 66.5% respectively (Figure 4B). Figure 4C showed that the body weights of the mice, and no obvious change was observed during treatment.

To assess whether the inhibition of tumor growth by Aea4- and Aea25 associated with the inhibition of FGFR1 in vivo, we studied the phosphorylation of FGFR1 in tumor tissue by western blot analysis. Figure 4D shows decreased expression of phosphorylated FGFR1 in the group both treated with in Aea4 and Aea25 compared to that of the untreated group, suggesting that Aea4 and Aea25 have a significant inhibitory activity against FGFR1. FGFR1 activation leads to the phosphorylation of ERK and Akt which are considered to be downstream signaling pathway of FGFR1, plays important role in the important roles in the proliferation and survival of cancer cells [25]. Thus the inhibition of phosphorylation of Erk and Akt in tumor samples was tested by immunohistochemistry. Similarly these compounds inhibited FGFR1 downstream ERK and Akt phosphorylation (Figure 4E). The expression of cell cycle and apoptosis related proteins was also detected, including Bcl-2 and Cox-2 (for anti-apoptosis) and cyclin D1 (for cell cycle) by immunohistochemisty. Aea4 and Aea25 effectively restrained the expression of Bcl-2, Cyclin D1 and cox-2 compared with model group (Figure 4E).

## DISCUSSION

The fibroblast growth factor receptor (FGFR) cascade plays crucial roles in tumor cell proliferation, angiogenesis, migration and survival [7, 10]. Accumulating evidence suggests that high expression of FGFR1 is closely related to the development of lung cancer especially in non-small cell lung cancers (NSCLC) [8, 9, 12, 26-28]. FGFR inhibition can reduce proliferation and induce cell death in a variety of in vitro and in vivo tumor models harboring FGFR aberrations, a growing number of research groups have selected FGFRs as targets for anticancer drug development [25, 29, 30].

FGFRs and other RTKs are highly conserved in the intracellular kinase domain where most small molecule inhibitors bind. As a result, it is difficult to develop highly specific inhibitors that distinguish between other RTKS and FGFR subtypes. Most of the FGFR1 inhibitors discovered to date are ATP competitive. They function by targeting the ATP binding domain of FGFR1. Unfortunately, this approach is becoming less effective, since ATP binding domain is conserved in all RTKs [13, 17]. Most of the FGFR1 inhibitors lack good kinase selectivity, its efficiency can be affected when high intracellular concentration of ATP exists, and they also result in a variety of side effects like nausea, weakness, hyperphosphataemia, and elevated blood pressure during clinical trial [15, 29]. PD173074 and SU5402 are recently reported ATP competitive FGFR1 inhibitors which failed to enter Phase II clinical trials due to their high toxicities [15]. To minimize the side effects of targeting FGFRs, non-ATP competitive FGFR1 inhibitor may have substantial benefits [14, 16]. Therefore, the exploration of novel inhibitors with better kinase selectivity has attracted extensive attention in recent years [13, 16, 31].

NDGA, a naturally occurring polyhydroxyphenolic compound has been previously characterized to have potential effects on cancer cell proliferation, apoptosis, and differentiation, with the ability to inhibit signaling by activated FGFR3, including kinase activation and downstream signaling [18]. In the present study, we firstly found that NDGA could inhibit FGFR1 activation, with a lower IC_50_ value than that against FGFR3. This indicates that NDGA may be a FGFR1 inhibitor and could be a leading compound to look for new FGFR1-inhibiting molecules. Subsequently, we designed and synthesized a series of novel NDGA analogues with the structure framework of bisaryl-1,4-dien-3-one. We found that out of 156 synthetic derivatives, Aea4 and Aea25 showed a significant selective inhibitory activity towards FGFR1 compared to the other targets such as FGFR2, FGFR3, cMET, EGFR and KDR, PDGFR-β. These findings indicate that the refined structures of NDGA achieved a higher potency against FGFR1 inhibition.

After screening for specific FGFR1 inhibition, we explored the mechanism of Aea4 or Aea25 to inhibit FGFR1. Interestingly we found that the compounds Aea4 or Aea25 suppressed the FGFR1 in an ATP independent manner which was well evidenced by the caliper mobility shift assay (Figure 2A-C) and molecular docking study (Figure 2D-E). ARQ069 is the first reported molecule that inhibits FGFR1/2 in a non-ATP-competitive manner, and its efficacy is not affected by high intracellular concentrations of ATP [14]. In fact, crystal X-ray structural analysis showed that ARQ-069 also acts on the ATP-binding pocket, but it combines with an inactive ATP binding site (“DFG-OUT” conformation), inducing a conformation shift that is quite different from the active ATP-binding site (“DFG-IN” conformation) [14]. Subsequently, our lab reported two new compounds, A114 and A117, which also showed non-ATP-competitive inhibition against FGFR1 via forming the “DFG-OUT” conformation [20]. In this study, it was observed that Aea4 and Aea25 inhibited FGFR1 potently, selectively, and without competing with ATP (Figure 1B and 2A). Then, we performed a docking study of Aea4 and Aea25 with FGFR1 kinase domain. The results showed that Aea4 and Aea25 may possess a similar FGFR1-binding mechanism when compared to ARQ069. The phenyl ring of the hydrophobic residue, F489, takes a downward movement under the interactions with Aea4/Aea25 and then leads to the stabilization of the inactive conformation of FGFR1 (Figure 2D-2E). The difference in the structure of the inactive form of FGFR1 may contribute to the selectivity of Aea4/Aea25. Thus, Aea4 and Aea25 represent a new kind of lead structure that inhibits FGFR1 in a ATP-independent manner.

At the cellular level, it was found that Aea4 and Aea25 dose-dependently inhibited the bFGF-mediated phosphorylation of FGFR1 in both FGFR1-overexpressing HEK293 cells (Figure 1C) and H460 cells (Figure 3A). Inhibition of ERK phosphorylation is consistent with inhibiting FGFR1 signaling pathway. Thus, the data further confirm that these compounds specifically target FGFR1.

A series of research have proved that FGFR1 was amplified in more than 10% of non-small cell lung cancers, and FGFR1-mediated signals pathway contribute to the growth, survival, and migration of NSCLC cells [8, 9, 32, 33]. Pharmacological inhibition of FGFR1 by small molecules could suppress the cell proliferation and induce apoptosis in NSCLC cells [12, 34]. Hence, next we moved our research to study the anti-tumor activity of Aea4 and Aea25 in NSCLC H460 cell line. As shown in Figure 3B-3F, Aea4 and Aea25 reduced the cell survival and increased the apoptosis in H460 cell line.

We further studied the anti-tumor activity of Aea4 and Aea25 in H460 xenograft mouse model. Treatment Aea4 or Aea25 resulted in reduction in both tumor volume and weight (Figure 4A-B), accompanied with a decrease in phosphorylated FGFR1 in tumor tissues of compound-treated mice. Suppression of key downstream signaling pathways, such as AKT and ERK, results in cell growth arrest and death [35]. With consistent to the above findings, immunohistochemistry analysis of tumor tissue treated with Aea4 or Aea25 revealed that inhibition of FGFR1 produces a subsequent reduction in phosphorylation of ERK/Akt and the anti-apoptotic protein Bcl-2, indicating that the apoptotic effects of Aea4 or Aea25 treatment result directly from an inhibition of the cell survival pathway regulated by the FGFR1. This was also well supported by the reduced expression of cyclin D1 in the treated group. The anti-proliferative effect of Aea4 or Aea25 on H460 tumor was associated with G1 cell-cycle arrest. Further, elevated level of Cox-2 has been implicated in angiogenesis, tumor growth and invasion [36, 37]. Treatment with Aea4 or Aea25 showed reduced expression of Cox-2 than compared to the untreated tumor group. Thus, all together we believe that the FGFR1-inhibitory actions of non-ATP dependent Aea4 and Aea25 inhibitors contribute greatly to its in vivo anticancer properties. At the same time, we observed that Aea4 and Aea25 exhibited high safety *in vivo* (Figure 4C). As already known, the ATP-competitive inhibitory mode leads to the biggest problems (toxicity and side effects) of current FGFR1 inhibitors [13, 29]. Our findings of Aea4 and Aea25 indicated that the non-ATP-competitive FGFR1 inhibition might be a new cancer therapeutic alternative with much lower toxicity *in vivo*.

In summary, this study demonstrated two novel, non-ATP-competitive inhibitors of FGFR1 kinase, i.e., Aea4 and Aea25, both of which exhibited good anti-tumor activity *in vitro* and *in vivo*. This study also presented a new leading structure for the design and development of non-ATP-competitive FGFR1 inhibitors. Aea4 and Aea25 should be considered to be developed as useful candidates for treating NSCLC and deserves additional study.

## MATERIALS AND METHODS

### Cell lines, compounds and reagents

Human lung carcinoma cell line NCI-H460 was purchased from ATCC (*Manassas*, VA). FGFR1-overexpressing 293 cells were kindly gifted by the Institute of Materia Medica at Xi'an Jiaotong University, China. Both of these two cell lines were incubated in 1640 medium (*Gibco*, Eggenstein, Germany) supplemented with 10% FBS (*Gibco*, Eggenstein, Germany), 100 U/mL penicillin, and 100 mg/mL streptomycin at 37°C with 5% CO_2_. All experiments were carried out 24 h after the cells were seeded. 156 NDGA structural analogs were previously designed and synthesized in our laboratory. The general chemical structure of these compounds is shown in Figure 1A. Before the biological evaluation, the compounds were purified by re-crystallization or silica gel chromatography which attains purity more than 95%. The compounds used in *vitro* were dissolved in DMSO. For the vivo experiments, the compounds, lecithin and cholesterol were weighed at the ratio of 1:5:1, dissolved in a moderate amount of propylene glycol and then added aqueous solution containing 1% Tween 80 maintened at 60C (the volume ratio of water and propylene glycol is 5:1). Finally, after high pressure homogeneous for 5 min, water soluble compounds in solution at 2mg/ml were ready for further study. Recombinant FGFR1, FGFR2, FGFR3, c-MET, EGFR, VEGFR-2, and PDGFR-β kinase proteins were purchased from Carna Biosciences Inc. (*Kobe*, Japan). DMSO and ATP were purchased from Sigma (St. Louis, MO). Antibodies such as anti-p-FGFR1, anti-FGFR1, anti-p-AKT, anti-p-ERK, anti-ERK, anti-GAPDH, anti-Actin, anti-Bcl-2, anti-Cyclin D1, anti-COX-2, anti-cleaved caspase-3, goat anti-rabbit IgG-HRP, and mouse anti-goat IgG-HRP were obtained from Santa Cruz Biotechnology (*Santa Cruz*, CA). PI/RNase staining buffer was purchased from BD Bioscience (*Franklin Lakes*, NJ). Recombinant bFGF protein was produced by our laboratory.

### *In vivo* anti-tumor study

Six-week-old BALB/cA nude mice (SPF degree, 6–8 weeks old and weighing18–22 g) were purchased from Animal Center of China Pharmaceutical University (Nanjing, China). All the animals were housed in specific pathogen-free (SPF) level laboratory with a 12 h light and dark cycle, and provided sterile food and water ad libitum. The mice were acclimatized for one week before being used for experiment. All the procedures were in strict accordance with the Wenzhou Medical University Policy on the Use and Care of Laboratory Animals (Wenzhou Medical University Policy and Welfare Committee, Document ID: WMU-2011-AP-0013). For in vivo studies, xenograft tumors were generated by inoculating harvested H460 cells mixed with Matrigel at a proportion of 1:1. Briefly, 2 × 10^6^ cells in 200 μL PBS cells were injected intraperitoneally on the right flank of the mice. After two days of H460 inoculation, the mice were treated intraperitoneally (i.p) with the water-soluble preparation of compound Aea4 or Aea25 in PBS at a dosage of 3 mg/kg/day for 28 days, and the control group were treated with liposome vehicle in PBS (n=10). The tumor volume was calculated using a microgauge according to the following equation: Tumor volume (mm^3^) = 1/2× (tumor length) × (tumor width)^2^. At the end of the experimental period all the animals were sacrificed by cervical decapitation and the tumor tissue was excised aseptically and the weight was recorded and used for the further study.

### Cell-free kinase activity assay

The activity of tyrosine kinase including FGFR1, FGFR2, FGFR3, EGFR, VEGFR-2, c-MET, and PDGFR-β were tested by Caliper Mobility Shift Assay on Caliper EZ reader (*Caliper Life Sciences*, MA) according to the instructions provided. Specific details of the method of this assay were described in our previous publication [20]. For each kinase assay, the ATP concentration used in the assay was set at the K_m_ value of the corresponding kinase. For the determination of IC_50_, the compounds were tested in duplicate at 10 concentrations, ranging from 5 nM to 100 μM. In the electrophoretic mobility shift assays, product accumulation was expressed as percentage conversion, product peak height/(product peak height + substrate peak height).

### ATP competitive inhibition assay

In the experiments for testing the relationship between the compounds and ATP, the concentration of the substrate was constant, while the concentrations of ATP were set at 4192, 2096, 1048, 524, 262, 131, 66, and33 μM. The global competitive inhibition fit for the compounds was performed based on percent conversion = (Vmax*X)/{km*[(1 + I/Ki)^n^] + X}, where X is the ATP concentration, and n is the Hill coefficient. Specific details of this method were presented in a previous report [20].

### Molecular docking

The docking simulation of Aea4 or Aea25 with FGFR1 was conducted with Tripos' molecular modeling package, i.e., Sybyl-2.0 (*Tripos, St. Louis, MO*). The crystal structure of FGFR1 was obtained from the Protein Data Bank (*PDB ID:* 3RHX). The ligand-binding groove on the proteins was kept rigid, whereas all torsible bonds of the ligands were set free to allow flexible docking to produce more than 100 structures. The final docked conformations were obtained when ligand poses with the lowest binding energy, and then the conformations were used to analyze the interaction mode between the ligand and its target.

### MTT Assay

MTT assay was used to evaluate the anti-proliferation activities of compounds against H460 cells. Cells were seeded in 96-well plates with 5000 cells per well, then cultured with Aea4, Aea25 and NDGA in concentration gradients for 72 hours. In addition, compounds were assayed for their anti-proliferative activities against H460 cell by time dependent MTT assay. Briefly, the H460 cell were seeded in 96-well plates with 5000 cells per well, then cultured with Aea4, Aea25 or NDGA (20μM) for 0, 12, 24, 36, 48 or 72 h, respectively. DMSO was used as the negative control. After treatment, the proliferation of the cells was determined by MTT assay.

### Western blot analysis

Cells or homogenated tumor tissues were lysated. The protein concentrations in all samples were determined by using the Bradford protein assay kit (*Bio-Rad, Hercules, CA*). The supernatant was run on 10% SDS-PAGE gel followed by being transferred to a PVDF membrane. After being blocked with 5% non-fat dry milk in TBST for 1.5h, the membrane was incubated with the primary antibody overnight followed by incubation with goat anti-rabbit IgG, HRP-linked antibody for 1 h. The blots were detected with an ECL detection kit (*Bio-Rad, Hercules, CA*) according to the manufacturer's procedure. The results were analyzed by Quantity One software to determine the relative band density ratio.

### Apoptosis analysis

Apoptotic cells were analyzed by flow cytometry with the use of Annexin V and propidium iodide (PI) staining (*BD Biosciences*, CA). In brief, H460 cells were seeded in 6-well plates with 1.2×10^5^ cells per well and allowed to grow overnight and treated with 10μM of compounds 24 h. After the treatment, cells were harvested with trypsin, washed twice with precooled PBS, and suspended in 1×loading buffer to achieve the concentration 1×10^6^cells per ml. Cells were stained with 5μl FITC Annexin V and 1μl PI, at room temperature for 15 min in the dark. The apoptosis cell rate was then measured with use of FACS calibur flow cytometry (*BD Biosciences*, CA).

### Immunohistochemistry

Excised tumor tissures were fixed in 10% neutral buffered formalin at room temperature for 18 to 24 h. The tissue was then dehydrated, embedded in paraffin and sectioned (5-μm sections) in a standard manner. All the slides were incubated with the primary antibodies. Sections were allowed to incubate in a humidified chamber for 1 h at room temperature. The slides were then rinsed in Tris buffer and incubated for 10 min at room temperature with a secondary antibody. After blocking the endogenous peroxidase activity with methanol and hydrogen peroxide, the slides were incubated with streptavidin-peroxidase complex. Diaminobenzidine was used as a chromogen to the protein. Sections were then rinsed in Tris buffer counterstained in hematoxylin, dehydrated, mounted and detected under microscope (*Nikon*, Japan).

### Statistical analysis

The values are expressed as mean ± SD and all the experiment data results were repeated for 3-5 times. Statistically significant differences between the sets of data were calculated using two-way analysis of variance (ANOVA) and Student's t-test using GraphPad Prism 5.0. Values of *P* < 0.05 were considered to be significant.
